# Thyroid hormone components are expressed in three sequential waves during development of the chick retina

**DOI:** 10.1186/1471-213X-8-101

**Published:** 2008-10-14

**Authors:** Jeffrey M Trimarchi, Sanjiv Harpavat, Nathan A Billings, Constance L Cepko

**Affiliations:** 1 Department of Genetics, Harvard Medical School, 77 Avenue Louis Pasteur, Boston, MA 02115, USA; 2 Texas Children's Hospital, Houston, TX 77030, USA; 3 Department of Genetics, Harvard Medical School, 77 Avenue Louis Pasteur, Boston, MA 02115, USA; 4 Department of Genetics and Howard Hughes Medical Institute, Harvard Medical School, 77 Avenue Louis Pasteur, Boston, MA 02115, USA

## Abstract

**Background:**

Thyroid hormone (TH) is an important developmental regulator in many tissues, including the retina. TH is activated locally via deiodinase 2 (Dio2), and it is destroyed by deiodinase 3 (Dio3). The TH receptors, TRa and TRb, mediate TH activity through hormone and DNA binding, and interactions with transcription regulators.

**Results:**

In the current work, the expression of these TH components was examined in the chick retina over time. Three waves of expression were characterized and found to be correlated with critical developmental events. The first wave occurred as progenitor cells began to make photoreceptors, the second as some cell types adopted a more mature location and differentiation state, and the third as Müller glia were generated. The cell types expressing the components, as well as the kinetics of expression within the cell cycle, were defined. TRb expression initiated during G2 in progenitor cells, concomitant with NeuroD and Otx2, which are expressed in early photoreceptor cells. TRb was expressed in photoreceptor cells for several days and then was reduced in expression level, as the expression of Crx, a later photoreceptor gene, became more evident. Dio3 was expressed throughout the cell cycle in progenitor cells. TRa was in most, if not all, retinal cells. Dio2 appeared transiently in a ventral (high) to dorsal gradient, likely in a subset of photoreceptor cells.

**Conclusion:**

Multiple TH components were expressed in dynamic patterns in cycling progenitor cells and photoreceptors cells across the developing chick retina. These dynamic patterns suggest that TH is playing several roles in retinal development, both within the cycling progenitor cells and possibly with respect to the timing of differentiation of photoreceptor cells.

## Background

TH controls normal development on a global scale, as evidenced by its central role in processes as diverse as amphibian metamorphosis and human cretinism [1-3]. While these roles have been known for some time, the mechanism(s) by which the hormone coordinates such events is still under active investigation. Considerable attention has focused on the hormone's regulation of the TRs, which are members of the thyroid/steroid nuclear receptor superfamily that bind DNA and activate or repress transcription [4,5]. TRs recruit activators such as the SMART complex, or repressors such as the HDACs, and alter the transcription of a variety of genes [6,7].

Equally important to the TRs are the deiodinases, which are enzymes that modify the prohormone secreted by the thyroid gland [8]. Deiodinases are expressed at different times by different tissues, and are thought to control the amount of active hormone available locally in a tissue [9,10]. Some deiodinases, such as Deiodinase 2 (Dio2), have 5'- outer ring (5'-) catalytic activity, clipping the 5'-iodine off of the T4 prohormone to create T3. T3 is the preferred ligand for TRs. Other deiodinases, such as Deiodinase 3 (Dio3), have 5- inner ring (5-) catalytic activity, removing the 5-iodine from either the T4 prohormone or the T3 active hormone, and thereby inactivating them. The inactive products do not bind with high affinity to the hormone binding site of the TRs. Deiodinase 1, a deiodinase not traditionally found in the nervous system, can have both 5'D and 5D activities.

As a first step in understanding how TH affects tissue development, the different TRs and deiodinases in a tissue must be identified. Previous work has shown that TH components are expressed at critical times during a particular tissue's development. For example, in the development of the murine inner ear, TH activating enzyme is necessary for proper inner ear development. Its expression peaks within a 2-day window, after which expression sharply declines [11]. Similarly, in the rodent heart, TR controls cardiac gene expression during the critical switch from embryonic to neonatal life [12].

Another tissue in which TH action has been studied is the retina. The retina has numerous advantages as a model tissue for developmental studies. It has a well-studied sequence of neural cell production, during which 7 different cell types are produced in an overlapping order [13,14]. Early reports identified three thyroid receptors, TRa, TRb0, and TRb2, in the developing chick retina using ISH [15-17]. TRb2 was found in the photoreceptor layer, TRa was observed in the progenitor layer, and TRb0 showed weak signal during later stages in the inner nuclear layer. Subsequent to this study, other investigators have used a hypothyroid rodent model to show that TH is important for retinal morphology [18], and in the amphibian retina, TH promotes proper morphology and proliferation [19,20]. In salmonid fish, TH appears to regulate the death and reappearance of UV cones during smoltification, a process similar to amphibian metamorphosis [21-23].

In addition to these studies, TH has been investigated with respect to its role in rodent photoreceptor development. Early *in vitro *studies implicated TH as a modulator of photoreceptor genes [24,25], and characterization of a TRb2 knock-out mouse showed that TRb2 is indeed involved in proper cone photoreceptor differentiation [26]. Without TRb2, cone photoreceptors expressed the short wavelength opsin (S-opsin) gene earlier than normal, and failed to express medium wavelength opsin (M-opsin). It is unclear whether this represents a fate change, from one cone cell type to another, or misregulation of the opsin genes.

We were intrigued by the number of processes controlled by TH, and looked to the chick retina for further insight into the processes regulated by TH. The chick retina is much larger than the rodent retina, thereby revealing spatiotemporal gradients of development more readily. We identified the expression patterns of the TRs as well as the deiodinases that activate and inactivate the hormone locally. The results show that TH components are expressed dynamically during development, in three sequential waves that spread across the retina in a central to peripheral pattern. The first wave marks the transition between cells that produce only mitotic daughter cells to cells that can produce neurons. The second wave marks a transition for some types of immature postmitotic cells to their more mature location and differentiation state. The third wave is correlated with the production of Müller glial cells. Furthermore, using dissociated cell ISH (DISH), we identified the cell types that express the TH components, and the kinetics of expression relative to the cell cycle. Together, these data suggest that TH plays multiple roles in chick retinal development, including a role within progenitor cells and photoreceptor cells.

## Results

### The expression of TH components in three distinct time windows

**Figure 1:**
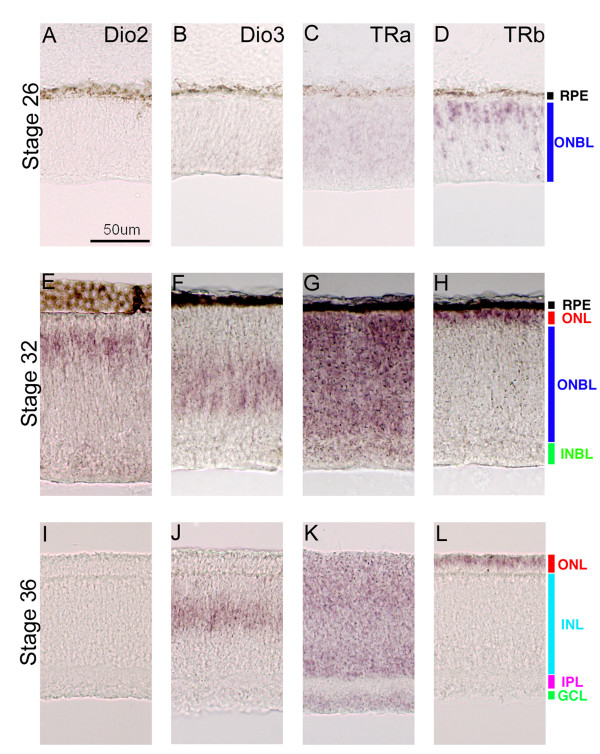
**Expression of TH components during chick development.** In situ hybridizations were performed on retinal cryosections cut in the coronal plane at three different stages of chick development (Stage 26 [A-D], Stage 32 [E-H], and Stage 36 [I-L]). The probes used were Dio2 (A, E, I), Dio3 (B, F, J), TRa (C, G, K), and TRb (D, H, L). A representative scale bar is shown.

Using section ISH, three discrete developmental stages were characterized for the expression of various TH components (Figure 1). These timepoints were separated by approximately 2 days, as developmental events occur first in the center, and then in the periphery, with the interval increasing as the retina grows larger [27-29]. The first timepoint during which the expression of the three components was examined was HH Stage 26. During this stage, TRa was observed throughout the retina, although at low levels (Figure 1C). TRb was observed in cells that were located throughout the outer neuroblastic layer (ONBL) of the retina, with more intense staining observed on the scleral side, near the RPE, where the photoreceptors will eventually reside (Figure 1D). The TRb in situ probe used in this study encompassed the common 3' end of both TRb0 and TRb2 and therefore did not distinguish between the two isoforms [16]. However, other experiments with probes made from the small N-terminal region specific to TRb2 showed similar, albeit lighter, staining [17]. Neither Dio2, the deiodinase that activates TH in the chick nervous system nor Dio3, the deiodinase that inactivates thyroid hormone, was detected in the retina at HH Stage 26 (Figure 1A, B).

During the second time period, HH Stage 32, the expression of all four TH components was observed (Figure 1E–H). TRa was broadly expressed throughout the developing retina (Figure 1G). Unlike TRa, the expression of TRb was restricted to the future photoreceptor layer, in a narrow band of cells abutting the RPE (Figure 1H). At this second developmental stage, the punctate TRb staining observed in cells distributed throughout the ONBL at HH Stage 26 had disappeared (compare Figure 1D with 1H). Dio3 was seen in cells in the center of the ONBL, where retinal progenitor cells reside [27,30] (Figure 1F and data not shown). Finally, Dio2 expression appeared in the retina, in a layer of cells one or two cell bodies below the RPE (Figure 1E). This cell layer is most closely associated with photoreceptors.

The third timepoint, HH Stage 36, showed lighter expression of the TH components, relative to the levels during the second time period (Figure 1I–L). Again, TRa was detected throughout the retina, in a similar expression pattern as at earlier time periods (Figure 1K), although at lower levels. TRb expression was also reduced, but still seen tightly abutting the RPE in a photoreceptor pattern (Figure 1L). Dio3 expression was observed in the center of the ONBL, where the few remaining progenitor cells at these late stages would be located [27](Figure 1J). Finally, just as in the first time period, Dio2 was not detectable by ISH in the retina at this later stage (Figure 1I).

### The first time period correlates with early neurogenesis

**Figure 2:**
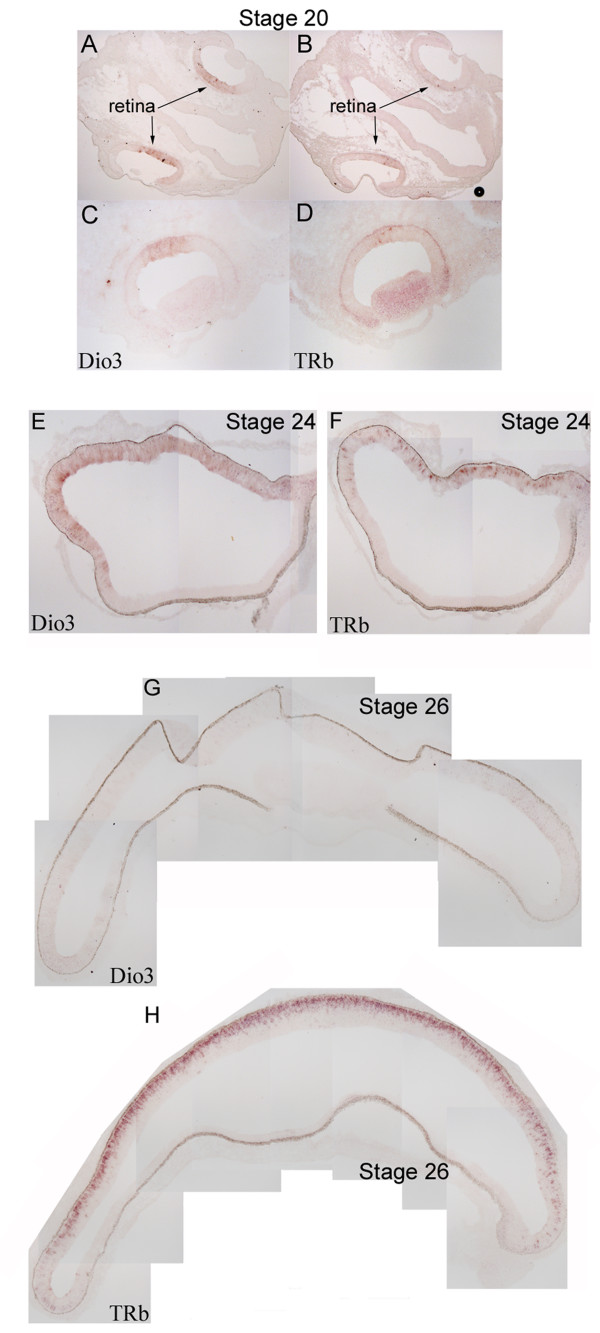
**Expression of TH components during early chick development.** In situ hybridizations were performed on serial cryosections cut in the coronal plane at three different stages of chick development (Stage 20 [A-D], Stage 24 [E, F] and Stage 26 [G, H]) for Dio3 (A, C, E, G) and TRb (B, D, F, H).The images in C and D are more centrally derived sections from an independent Stage 20 chick.

To better characterize the dynamic expression patterns of the TH components, the expression patterns were further examined at several early HH stages. During HH Stages 20–26, TRb expression appeared as an enlarging wave, starting from the center and expanding to the periphery, sometimes overlapping the expression domain of Dio3 (Figure 2A–H). At Stage 20, Dio3 expression was observed centrally, overlapping the domain containing a small number of TRb-positive cells (Figure 2A–D). At Stage 24, Dio3 and TRb expression were both more robust, having now begun to spread more peripherally from the central portion of the retina (Figure 2E, F). The expression domain of both genes overlapped, with Dio3 expression sometimes being observed slightly ahead (i.e. more peripherally) of the TRb domain (Figure 2C–F). By Stage 26, Dio3 expression had waned significantly, with only faint expression observed in the periphery (Figure 2G, H). In contrast, at Stage 26, TRb expression was robust both centrally and peripherally, and was not yet resolved to the photoreceptor layer, as was observed at later stages.

**Figure 3:**
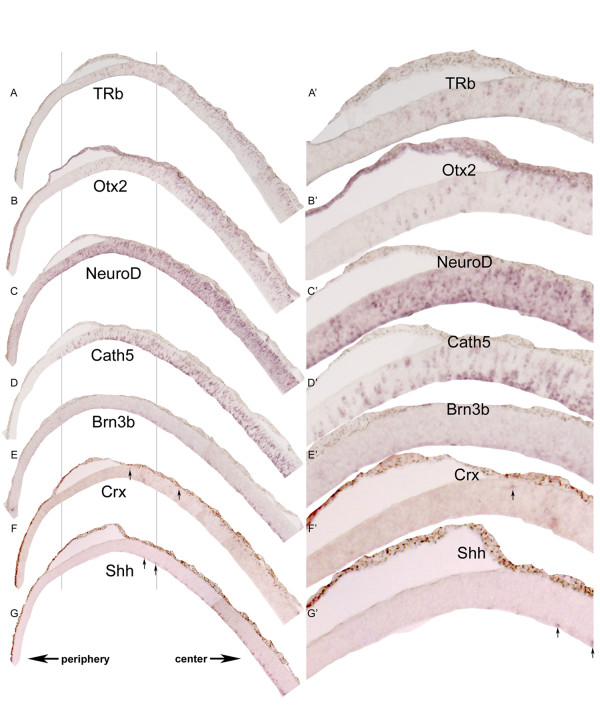
**Expression of TH components and early neurogenesis markers.** Section ISH composites of Stage 26 chick retinal cryosections cut in the coronal plane using the following probes: TRb (A, A'), Otx2 (B, B'), NeuroD (C, C'), Cath5 (D, D'), Brn3b (E, E'), Crx (F, F'), and Shh (G, G'). The arrows at the bottom of the low magnification sections indicate the central and peripheral retina. Zoomed-in views (using Adobe Photoshop) from the indicated portions of each section are shown on the right. Arrows point toward positive cells for the indicated probes.

Since the center-to-periphery TRb wave resembled that seen with the neurogenic genes Otx2 [31], NeuroD [32], and Cath5 [33], serial section ISH was used to see if the different expression patterns were correlated (Figure 3). Otx2 and NeuroD were expressed in cells scattered throughout the ONBL, with a higher concentration of labeled cells located near the scleral side (Figure 3A–C'). Both genes are thought to mark early photoreceptor cells, as well as other cell types [31,32,34-36]. Cath5, on the other hand, was expressed in a bilayered pattern, present in cells in the ganglion cell layer as well as in cells that have not been defined, but which overlap the developing photoreceptor layer (Figure 3D, D') [33]. The expression pattern of NeuroD and Cath5 appeared to extend slightly more toward the peripheral retina than TRb and Otx2, although all 4 genes were similar in the extent of their central-peripheral expression (Figure 3A–D). These data suggest that TRb expression is correlated with a more general wave of neurogenesis spreading across the retina at early stages.

TRb expression also was compared to the expression of Brn3b, Crx, and Shh, three genes marking the early differentiation of neurons, which also occurs in a central-to-peripheral pattern. In mouse, Brn3b marks early ganglion cells [37-39] and Crx marks early photoreceptor cells [40]. Shh is expressed by ganglion cells in the chick [41], and possibly in other cell types as well. In zebrafish, ganglion cells and a subset of amacrine cells express Shh [42], and in mouse, there is a similar pattern of expression [43], and there is a history of expression of Shh in a subset of photoreceptors, likely cone photoreceptors [44]. At Stage 26, the location of cells expressing Brn3b, Crx, and Shh expression was similar with respect to the central/peripheral gradient, within the expression domain located more centrally then TRb (compare Figure 3A, A' with 3E–G').

### The second time period corresponds to a wave of maturation of some cell types

**Figure 4:**
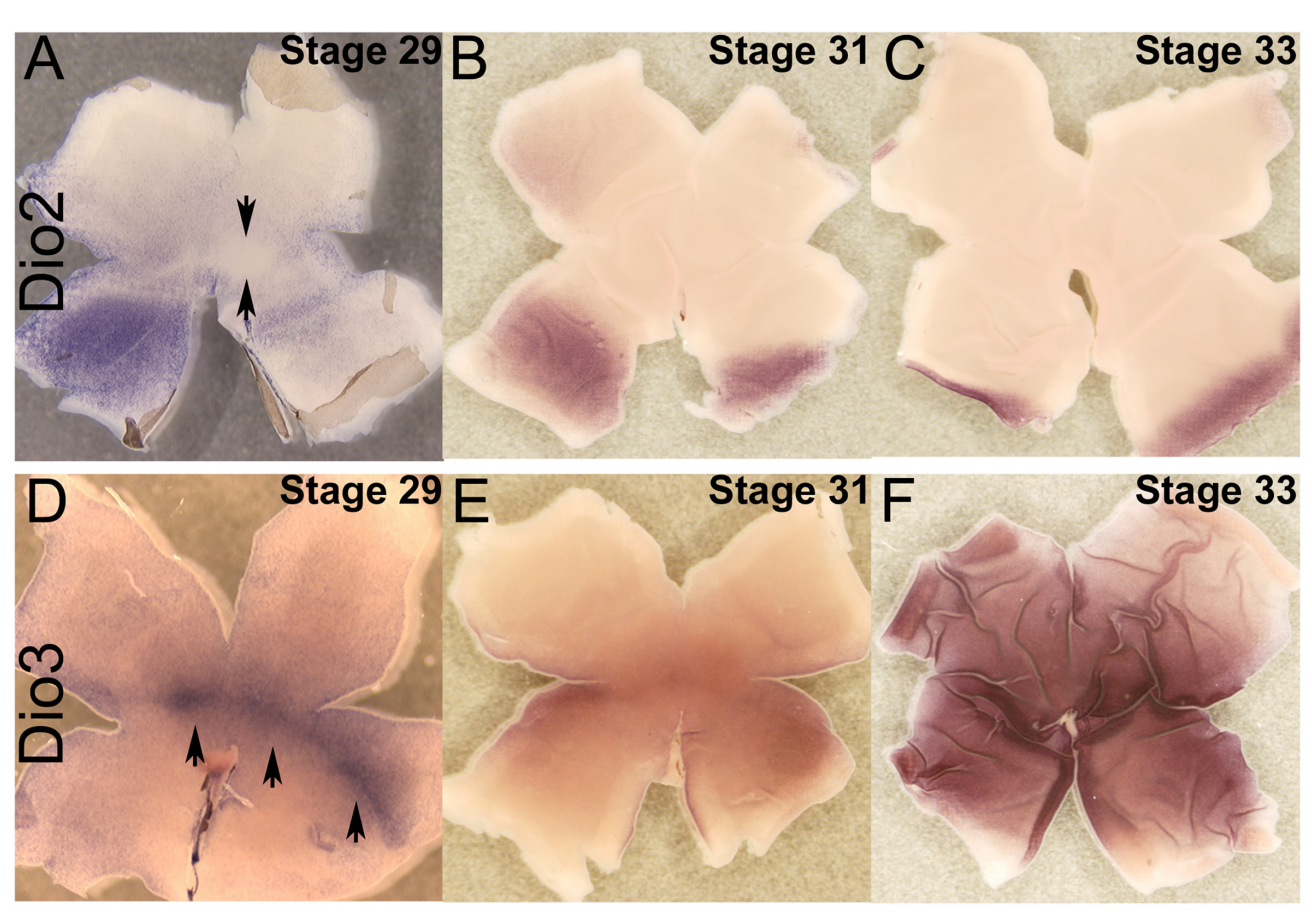
**Expression of TH components during the second wave.** In situ hybridizations were performed on flat mounted retinas at three different stages of chick development (Stage 29 [A, D], Stage 31 [B, E] and Stage 33 [C, F]. The probes used were Dio2 (A-C) and Dio3 (D-F). The dorsal side of the retina is at the top of the flatmount and the ventral side at the bottom, while anterior (nasal) is toward the left and posterior (temporal) is toward the right. Arrows in (A) indicate the spot that is devoid of Dio2 staining. The arrows in (D) identify the stripe pattern of Dio3 expression at Stage 29.

**Figure 5:**
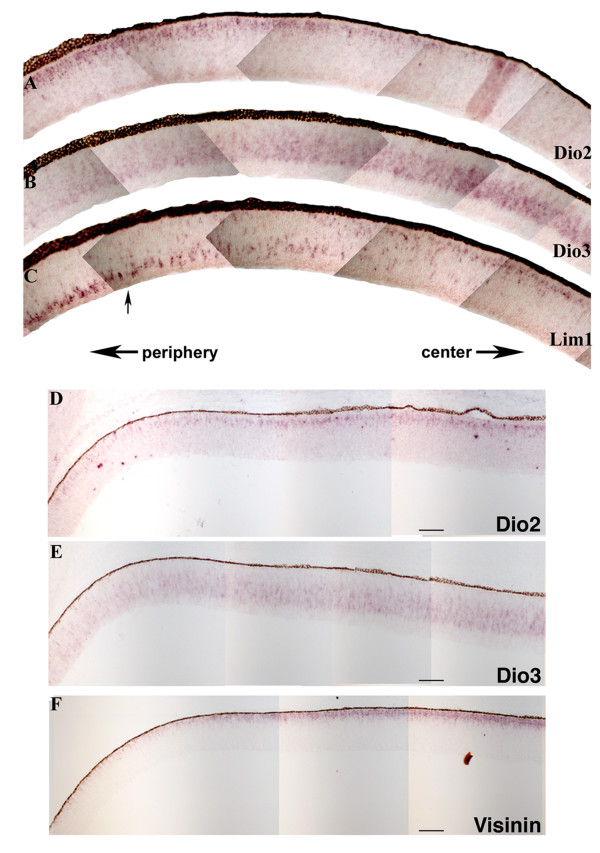
**Expression of TH components and mature neuronal markers.** In situ hybridizations were performed on serial cryosections cut in the coronal plane and collected from near the central retina (level of the optic nerve) at Stage 31. Composites of the ISH are shown for Dio2 (A), Dio3 (B) and Lim1 (C). The arrow in C indicates the location of the beginning of Lim1 migrating cells. Composites of ISH on a second set of serial sections at Stage 31 are shown for Dio2 (D), Dio3 (E) and Visinin (F). Arrows under panel C indicate the center and peripheral portions of all of the retinas in the figure. Representative scale bars for 100 μm are shown.

In a second time period, HH Stages 29–33, a second wave of TH components involving Dio2 and Dio3 swept across the retina (Figure 4). Using flat mount ISH, Dio2 expression was observed at Stage 29 as heavy ventral staining, some dorsal staining, and an absence of expression in a spot and stripe centered on the dorsoventral border (Figure 4A, arrows). Over the next few stages, Dio2 expression was confined in narrower stripes toward the periphery (Figure 4B, C). In a nearly complementary pattern, Dio3 started at Stage 29 as a spot and stripe centered on the dorsoventral border (Figure 4D, arrows). Over the next few stages, Dio3 expression expanded dorsally and ventrally to occupy the entire retina (Figure 4E, F). ISH performed on serial sections of the peripheral retina at HH Stage 31 showed that the ebbing Dio2 and expanding Dio3 expression patterns were nearly complementary (Figure 5A, B).

During HH stages 20–26, there was a correlation of expression of TH components and genes involved in neurogenesis and differentiation (Figure 3). It was of interest to determine whether the second wave of expression of TH components also was correlated with other genes. The first candidate examined was Lim1, which has been shown to mark horizontal cells as they move from the vitreal side to their final scleral position during development [45]. On serial sections, the interface between Dio2 and Dio3 occurred in the same place as Lim1 cell migration (Figure 5A–C).

The Dio2/Dio3 wave also paralleled changes the photoreceptor marker, Visinin (Figure 5D–F). At the Dio2/Dio3 wavefront/transition, Visinin expression became more organized, transitioning from a weak diffuse pattern to a more intense band in the maturing photoreceptor layer (Figure 5D–F). Hence, the transition in the expression of Dio2 and Dio3 occurred along with the transition of a couple of genes to their mature expression patterns.

### The third time period corresponds to the disappearance of progenitor cells

**Figure 6:**
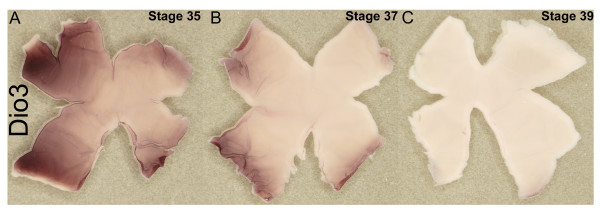
**Expression of Dio3 during the third wave.** In situ hybridizations were performed using a Dio3 probe on flat mounted retinas at three different stages of chick development: Stage 35 (A), Stage 37 (B), and Stage 39 (C). The dorsal side of the retina is at the top of the flatmount and the ventral side at the bottom, while anterior (nasal) is toward the left and posterior (temporal) is toward the right.

**Figure 7:**
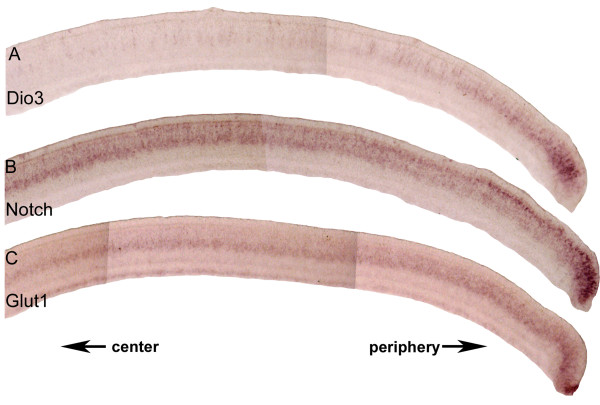
**Expression of Dio3, Notch1, and Glut1 during the third wave.** In situ hybridizations were performed on serial cryosections cut in the coronal plane at Stage 37 for Dio3 (A), Notch (B) and glucose transporter 1 (Glut1) (C). Arrows indicate the direction of the center and periphery of the retina.

In a later time period, HH Stages 35–39, a third wave involving Dio3 swept across the retina (Figure 6). During this time period, Dio3 expression ceased, first from the center (Figure 6A) and then in the periphery (Figure 6C). Similar to the previous 2 waves of expression of TH components, this period showed a correlation with the expression of genes involved in retinal development. On serial sections, Dio3 was expressed throughout the area occupied by progenitor cells as defined by the expression of Notch (Figure 7A, B). In more central areas, where Dio3 expression was absent, Notch appeared in the layer that seemed to overlap with the expression of a glucose transporter (Glut1) (compare Figure 7B and 7C), in a pattern consistent with Müller glia cell expression [46,47]. Birthdating studies have shown that Müller glia cells are among the last born of retinal cell types [14], and thus this transition appears with the end of proliferation.

### TRb is expressed in early photoreceptor cells

Previous data concerning TRb expression in the murine retina [17,26,48], along with the location of the ISH signal in the scleral region of the chick retina (Figure 1H, L), suggested that TRb is expressed by photoreceptor cells. However, the scleral region of the retina also harbors cells in M phase, as well as cells that are newly postmitotic, but not photoreceptor cells. In order to define the cell type(s) expressing TRb, dissociated cell ISH (DISH) was carried out. Probes for cell type-specific markers along with probes for TH components were used. In addition, to investigate whether TH components were expressed in mitotic cells, newly postmitotic cells, or postmitotic cells that had not recently exited, and were thus further along in their differentiation, embryos were labeled with [^3^H]-thymidine *in ovo*. DISH was used to detect expression of one or two genes in cells labeled with [^3^H]-thymidine (Additional Files 1, 2, 3, 4, Tables 1, 2).

**Table 1:** The kinetics of coexpression of TH components and marker genes at Stage 31

**gene 1**	**gene 2**	**% gene 1^+^**	**% gene 2^+^**	% gene 1^+^/gene 2^+^*	**% gene 2^+^/gene 1^+^**	**% [^3^H]^+^**	**% of gene 1^+ ^cells that are [^3^H]^+^**	**% of gene 2^+ ^cells that are [^3^H]^+^**	**% of [^3^H]^+ ^cells that are gene 1^+^**	**% of [^3^H]^+ ^cells that are gene 2^+^**	**% of [^3^H]^+ ^cells that are both gene 1^+ ^and gene 2^+^**
otx2	TRb	36.4	15.2	41	97.8	14.9	9.9	2.2	24.2	2.2	2.2
dio2	cath5	1.2	10.2	30	3.4	17.5	0	1.1	0	0.7	0
cath5	neurod	16.5	28.3	28.4	16.5	15	2.5	13	2.7	24.3	2.7

Cells were labeled in ovo with [^3^H]-thymidine for 1 hour, and tissue was harvested and dissociated at the indicated times. Double DISH for the indicated genes and autoradiography for [^3^H] were carried out on the dissociated cells. Cells were quantified for labeling by a single gene probe, both gene probes, and labeling with [^3^H].

**Table 2:** The kinetics of coexpression of TH components and marker genes at Stage 32

**gene 1**	**gene 2**	**% gene 1^+^**	**% gene 2^+^**	% gene 1^+^/gene 2^+^*	**% gene 2^+^/gene 1^+^**	**% [^3^H]^+^**	**% of gene 1^+ ^that are [^3^H]^+^**	**% of gene 2^+ ^that are [^3^H]^+^**	**% of [^3^H]^+ ^that are gene 1^+^**	**% of [^3^H]^+ ^that are gene 2^+^**
dio2	cath5	2.3 ± 0.5	9.4 ± 0.4	17.4 ± 4.5	4.0 ± 1.0	17.8 ± 0.9	0	2.0 ± 0.5	0	1.0 ± 0.3
dio2	TRb	3.2 ± 0.4	7.4 ± 1.1	14.3 ± 4.5	5.8 ± 1.3	16.4 ± 1.2	0	7.3 ± 0.9	0	3.3 ± 0.6
dio2	otx2	2.9 ± 0.1	30.5 ± 1.4	91.9 ± 4.2	8.7 ± 0.8	17.5 ± 1.0	3.2 ± 3.2	14.8 ± 0.3	0.5 ± 0.5	25.9 ± 1.8
dio2	dio3	2.9 ± 0.4	8.1 ± 0.9	0	0	16.4 ± 0.9	2.4 ± 1.2	42.8 ± 5.5	0.5 ± 0.2	20.8 ± 2.1
dio2	crx	3.3 ± 0.4	13.5 ± 1.0	70.9 ± 11.2	17.4 ± 3.3	17.8 ± 1.7	1.5 ± 1.5	0.6 ± 0.3	0.4 ± 0.4	0.5 ± 0.3
dio2	neurod	2.2 ± 0.2	23.6 ± 2.0	89.3 ± 2.2	8.5 ± 1.3	16.9 ± 1.1	2.1 ± 2.1	11.6 ± 1.1	0.3 ± 0.3	16.1 ± 1.0
TRb	neurod	8.7 ± 0.5	26.8 ± 0.6	95.5 ± 1.4	31.1 ± 1.7	17.9 ± 0.4	6.2 ± 1.3	12.1 ± 1.1	3.1 ± 0.9	18.1 ± 1.7
TRb	cath5	7.5 ± 0.9	12.5 ± 1.1	10.1 ± 2.6	5.9 ± 1.4	17.5 ± 0.6	10.1 ± 0.7	1.0 ± 0.6	4.2 ± 0.2	1.8 ± 0.6
TRb	otx2	7.7 ± 1.2	26.8 ± 0.7	95.7 ± 1.3	27.5 ± 3.9	17.5 ± 0.7	9.0 ± 2.6	14.3 ± 2.1	3.8 ± 0.8	21.6 ± 2.1
TRb	crx	6.6 ± 0.2	13.5 ± 0.7	67.5 ± 5.5	33.1 ± 2.9	17.3 ± 0.6	13.8 ± 3.5	1.8 ± 1.0	5.3 ± 1.3	1.4 ± 0.7
dio3	crx	10.7 ± 0.4	12.9 ± 1.6	0	0	18.9 ± 0.8	46.6 ± 1.4	1.0 ± 0.7	26.3 ± 0.6	0.6 ± 0.4
dio3	TRb	9.6 ± 0.9	8.1 ± 0.4	1.1 ± 0.6	1.1 ± 0.6	17.9 ± 1.1	50.9 ± 5.7	9.8 ± 0.9	26.8 ± 1.3	4.5 ± 0.9
dio3	neurod	8.5 ± 0.9	24.8 ± 3.0	24.8 ± 4.6	8.5 ± 1.3	18.1 ± 1.1	45.4 ± 6.0	8.3 ± 0.8	21.1 ± 1.7	11.7 ± 2.6
dio3	otx2	8.5 ± 1.0	25.8 ± 2.1	44.1 ± 13.4	13.4 ± 2.4	17.0 ± 0.9	45.3 ± 2.8	10.5 ± 1.7	22.4 ± 2.0	16.1 ± 3.3

Cells were labeled in ovo with [^3^H]-thymidine for 1 hour, and tissue was harvested and dissociated at the indicated times. Double DISH for the indicated genes and autoradiography for [^3^H] were carried out on the dissociated cells. Cells were quantified for labeling by a single gene probe, both gene probes, and labeling with [^3^H].

* % of cells that express gene 1 that also express gene 2

**Figure 8:**
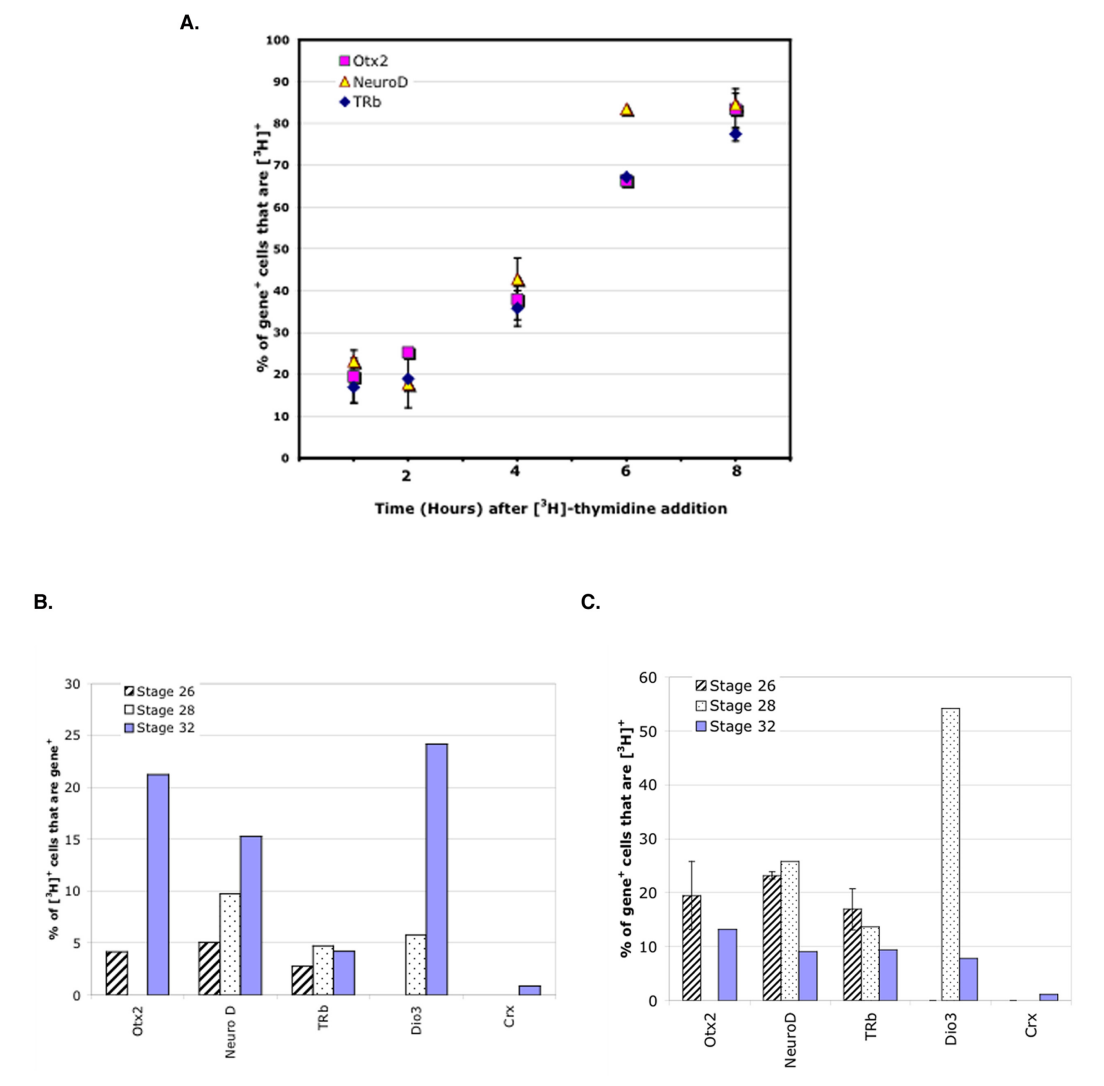
**DISH for TH components, early neurogenic markers and mature neuronal markers.** Cells were labeled in ovo with [^3^H]-thymidine, and tissue was harvested and dissociated at the indicated times. DISH for TRb, Otx2, Dio3, Crx, or NeuroD and autoradiography for [^3^H] were carried out on the dissociated cells. (A) The percentage of Otx2^+^, NeuroD1^+^, or TRb^+ ^cells that are also [^3^H] ^+ ^at increasing times after [^3^H]-thymidine addition at Stage 26. (B) The percentage of [^3^H]^+ ^cells, after a 1 hour labeling time, that were also Otx2^+^, NeuroD1^+^, TRb^+^, Dio3^+ ^or Crx^+ ^are shown for Stage 26, Stage 28 and Stage 32. (C) The percentage of Otx2^+^, NeuroD1^+^, TRb^+^, Dio3^+ ^or Crx^+ ^cells that were also [^3^H]^+ ^after a 1 hour labeling time are shown for Stage 26, Stage 28 and Stage 32.

A one hour pulse with [^3^H]-thymidine, followed by an immediate harvest, would be expected to label primarily cells in S phase, along with cells in the early part of G2 [27]. DISH for TRb applied to cells labeled with [^3^H]-thymidine for one hour showed that most of the TRb expressing cells were not co-labeled for [^3^H]-thymidine and thus were not in S phase at Stages 26, 28, or 32 (Figure 8, Additional Files 1, 2, Tables 1, 2, Additional File 4E–F). In addition, the majority of cells in S phase or early G2 at these stages did not express TRb (Figure 8, Additional Files 1, 2, Table 3, Additional File 4E–F). However, since a small fraction of cells, 2.8 ± 0.7%, labeled with [^3^H]-thymidine after one hour expressed TRb (Figure 8) it is likely that TRb turns on in some G2 cells. These data are in keeping with data from the ISH on tissue sections, in which the majority of TRb^+ ^cells were located in the area of G2 cells and photoreceptor cells (Figure 1D).

**Table 3:** The coexpression of TH components and known marker genes at Stage 30 and Stage 31

**stage**	**gene 1**	**gene 2**	**% gene 1^+ ^cells**	**% gene 2^+ ^cells**	**% of gene 1^+ ^cells that are also gene 2^+^**	**% of gene 2^+ ^cells that are also gene 1^+^**
30	crx	visinin	20.9	19.2	89.4	97.5
30	visinin	crx	17.1	17.3	95.2	93.8
30	dio2	cath5	1.1	16.3	85.7	5.8
30	cath5	dio2	13.5	0.6	4.3	100
30	visinin	TRb	16.7	14.6	60.9	69.6
30	TRb	visinin	17.6	16.3	70.3	75.9
						
31	neurod	cath5	22.5	9.5	12.4	29.3
31	neurod	TRb	20.4	6.9	30.1	89.3
31	otx2	cath5	28.6	10.4	6	16.5
31	TRb	cath5	8.7	12.3	10.3	7.2
31	TRb	crx	8.5	17.6	87.4	42.1

Cells were labeled in ovo with [^3^H]-thymidine for 1 hour, and tissue was harvested and dissociated at the indicated times. DISH for the indicated genes and autoradiography for [^3^H] were carried out on the dissociated cells.

Examination of [^3^H]-thymidine^+ ^cells at intervals after the initial addition of the label allows a determination of whether a gene is expressed in G2, M, or G1/G0, since injection of [^3^H]-thymidine *in ovo *results in continuous labeling with [^3^H]-thymidine [49,50]. DISH for TRb was performed on cells from Stage 26 or 28 embryos 1–10 hours after addition of [^3^H]-thymidine (Figure 8A, Additional Files 1, 2, Additional File 4E–H). These data show a higher percentage of cells that express TRb were labeled with [^3^H]-thymidine over time, resulting in 77.4 ± 1.6% (Additional Files 1, 2) of TRb^+ ^cells also being labeled with [^3^H]-thymidine by 8 hours at Stage 26, and 62.2% (Additional Files 1, 2) at Stage 28 by 10 hours. Since the cell cycle length is 10 hours at these stages [27], the majority of TRb^+ ^cells are within one cell cycle length of labeling. Given the location of TRb cells in the tissue section in the area of photoreceptor cells, the lack of S phase labeling for the majority of TRb^+ ^cells, the low numbers of TRb^+ ^cells in the G2 population (i.e. those labeled with [^3^H]-thymidine after 1–4 hours), most TRb^+ ^cells are likely newly postmitotic, and are likely to be photoreceptors. This was confirmed by double DISH using photoreceptor markers (Table 3, Additional File 3A–F).

As mentioned above, NeuroD and Otx2 are transcription factors that are expressed by newborn photoreceptors [31,32], and by other retinal cell types as well. The expression of Otx2 and NeuroD in the cell cycle was examined as described above, and was compared to the labeling of TRb (Figure 8, Additional Files 1, 2, 4). Cells that expressed NeuroD or Otx2 showed kinetics of labeling with [^3^H]-thymidine almost identical to the labeling kinetics of TRb^+ ^cells (Figure 8A). Double DISH at Stage 31 for TRb and NeuroD, or TRb and Otx2, revealed that 89.3% of all TRb^+ ^cells expressed NeuroD (Table 3), and 97.8% expressed Otx2 (Table 1, Additional File 3A–C). The expression of Otx2 and NeuroD was examined among the [^3^H]^+ ^cells labeled in a one hour exposure to [^3^H]-thymidine (Figure 8B, C). Of the [^3^H]^+ ^cells at Stage 31, 24.2% were Otx2^+^, and 24.3% were NeuroD^+^, while only 2.2% were TRb^+^. Thus, among the [^3^H]^+ ^cells that expressed Otx2, or NeuroD, only approximately one out of 10 also expressed TRb (Tables 1, 2). These data demonstrate that while the kinetics of onset of expression of Otx2, NeuroD, and TRb are similar, many more cells, particularly at the later stages examined, expressed NeuroD and Otx2 than TRb.

**Figure 9:**
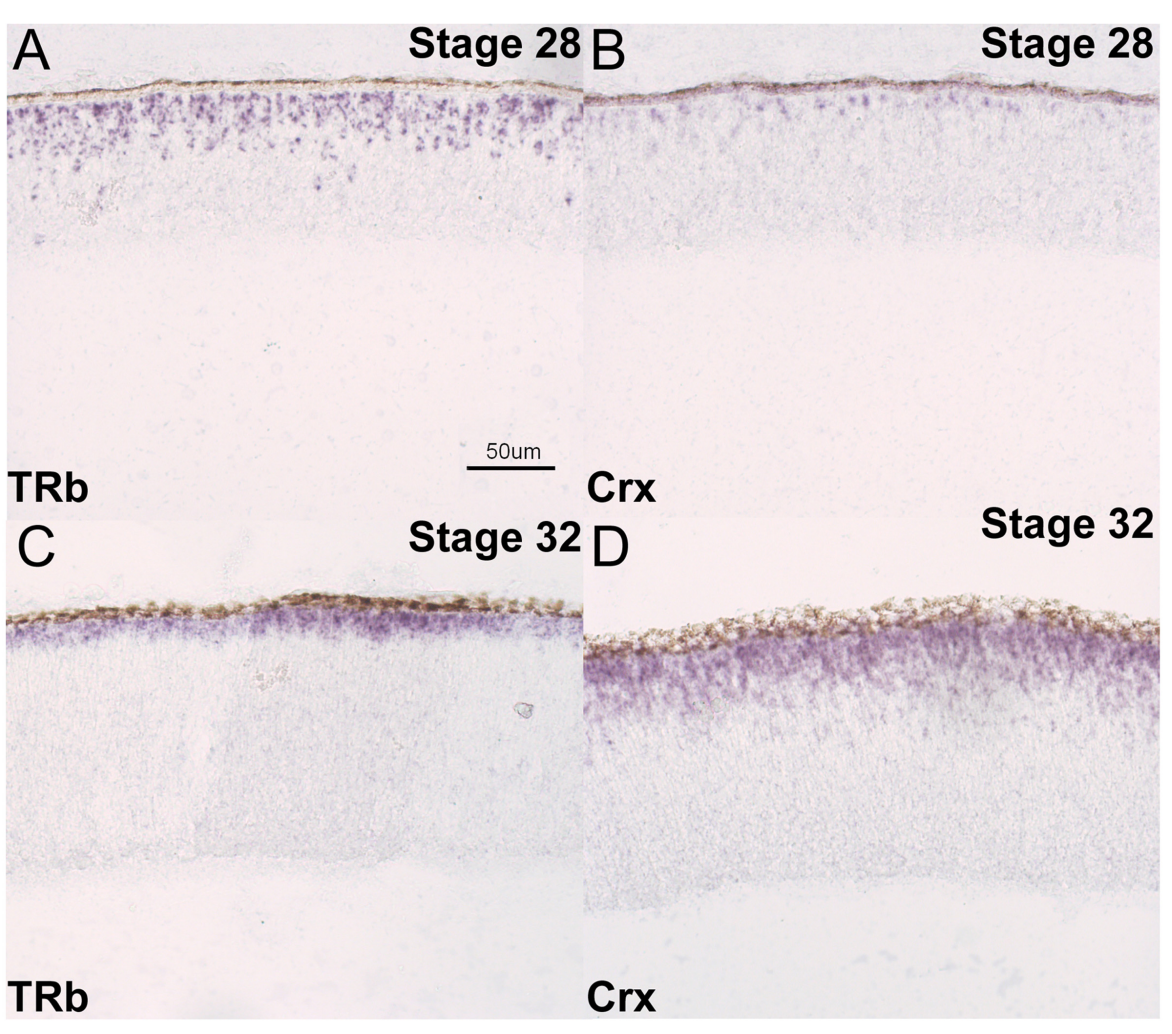
**Expression of TRb and Crx during chick development.** In situ hybridizations were performed on retinal cryosections cut in the coronal plane at two different stages of chick development (Stage 28 [A, B] and Stage 32 [C, D]). The probes used were TRb (A, C) and Crx (B, D). A representative scale bar is shown.

Co-expression of two other markers of photoreceptors, Visinin and Crx, along with TRb was examined by double DISH. Visinin is expressed exclusively by photoreceptors [28,51], and 70.3% of all TRb^+ ^cells expressed Visinin at Stage 30 (Table 3). The percentage of TRb^+ ^cells that expressed Crx was 87.4% at Stage 31 (Table 3) and 67.5% at Stage 32 (Table 2 and Additional File 3D–F). TRb and Crx co-expression was compared further. Crx can be considered a later photoreceptor marker compared to NeuroD and Otx2, since, at Stage 26, <0.1% of [^3^H] ^+ ^cells were Crx^+^; at Stage 28, <0.1% of [^3^H] ^+ ^cells were Crx^+^; and, at Stage 28, 4–8 hours after addition of [^3^H]-thymidine, only 1.0–2.2% of the [^3^H]^+ ^cells were Crx^+ ^(Additional Files 1, 2, 4I–L). TRb and Crx expression on section ISH displays an inverse relationship: before Stage 29. TRb was observed in a greater number of cells than Crx, whereas after Stage 29, the opposite was observed (compare Figure 9A and 9B to 9C and 9D). This pattern was confirmed using DISH. At Stage 28, TRb was expressed in approximately 9% of cells, and Crx was observed in 7% of all cells, whereas at Stage 32 TRb was mildly reduced to approximately 7%, while Crx increased to 14% of all cells.

The transient nature of the co-expression of TRb and Crx in differentiating photoreceptors was confirmed by double DISH with TRb and Crx. At Stage 29, 26% of TRb^+ ^cells expressed Crx, and 94% of Crx^+ ^cells expressed TRb (data not shown). At Stage 31, 87.4% of TRb cells expressed Crx, whereas 42.1% of Crx cells expressed TRb (Table 3). At Stage 32, 67.5 ± 5.5% of TRb cells expressed Crx, and 33.1 ± 2.9% of Crx cells expressed TRb (Table 2). This pattern is consistent with an earlier onset of TRb than of Crx in newly postmitotic photoreceptor cells, followed by a period of co-expression of TRb and Crx, and then a reduction in expression of TRb in photoreceptors, which retain expression of Crx. It also suggests that there is a greater interval between the onset of TRb and the onset of Crx at the later stages. The stability of expression of Crx among photoreceptors is reflected in the high co-expression of Crx and Visinin (approximately 90%) at Stage 30 (Table 3).

### Dio2 is expressed in a subset of photoreceptor cells

Dio2 expression also was investigated using DISH. Dio2 was not detected at Stage 26, but later was expressed in a small but increasing percentage of all cells: Stage 30, approximately 0.6%, Stage 31, approximately 1.2%, and Stage 32, approximately 3% (Tables 1, 2, 3). Double DISH showed that at Stage 32, almost all Dio2^+ ^cells expressed NeuroD (89.3%) and Otx2 (91.9%), while 70.9% expressed Crx and 14.3% expressed TRb (Table 2, Additional file 3G–L). At Stage 30, 85–100% of Dio2^+ ^cells expressed Cath5, but they likely turn it off as only 17% expressed Cath5 by Stage 32. These data suggest that Dio2 is present in photoreceptor cells, but in a subset that appears at a later time in development. With respect to its kinetics in the cell cycle, Dio2 was expressed later than Cath5, TRb, NeuroD, and Otx2 since Dio2 expression was almost never observed in S/G2 cells (Tables 1, 2, Figure 8B, C, Additional File 4Q–R). Combined with the section ISH results, Dio2 appeared in a subset of photoreceptors as they transitioned from newly postmitotic cells to later stages of differentiation.

### Dio3 is expressed in mitotic cells

Dio3, whose expression appeared to be in progenitor cells on section ISH, was also studied using DISH and [^3^H]-thymidine incorporation. At Stage 28, 54.2% of Dio3^+ ^cells were [^3^H]^+ ^after a one hour pulse (Additional Files 1, 2, 4M–N), and by 8 hours of [^3^H]-thymidine labeling, all Dio3^+ ^cells were labeled with [^3^H] (Additional File 4O–P). This is in contrast to the plateau of [^3^H]-thymidine labeling for populations that include postmitotic cells, such as NeuroD (84% at 8 hours) and TRb (67% at 8 hours). Dio3 was not present in cells that expressed Crx (0%), or Dio2 (0%), and only very few cells that expressed Dio3 also expressed TRb (1.1%), at Stage 32. However, many cells that expressed Dio3 also expressed Otx2 (44.1%) and NeuroD (24.8%) at Stage 32. Of these Dio3^+^Otx2^+ ^or Dio3^+^NeuroD^+ ^cells, 44.5 ± 4.3% and 57 ± 8.3% respectively of them were also [^3^H]^+ ^after a 1 hour pulse. These data indicate that cells that co-express Dio3 and Otx2, and/or NeuroD, are in S phase and/or early G2.

## Discussion

In this report, we show that TH components are expressed in three sequential waves that spread across the retina, and that these waves are correlated with expression changes in a number of developmentally important genes. Furthermore, we identify which cell types express which TH components, and determine their expression kinetics with respect to the cell cycle.

### Waves are a common theme in retinal development

In a diverse array of organisms, retinal development occurs in waves. The best example of this is in *Drosophila melanogaster*, in which the eye differentiates in a wave defined by the morphogenetic furrow [52]. Cells ahead of the furrow express genes involved in cell division and the undifferentiated state, whereas cells behind the furrow express cell differentiation markers as well as genes signaling terminal cell divisions. The phenomenon of genes expressed in differentiation waves also extends to vertebrate eye development. In zebrafish and *Xenopus*, a program of cell differentiation spreads across the early retina, from the center to the periphery, as exemplified by the expression of Shh [53,54]. Similarly, in the chick retina, the expression of neurogenesis markers has been observed to spread in an orderly fashion from the center to the periphery [29,55]. In *Drosophila*, there is a relationship between a nuclear hormone receptor, the ecdysone receptor, and hedgehog [56-58]. It appears that the relief of repression from ecdysone receptor occurs just anterior to the morphogenetic furrow, while hedgehog initiates expression within the MF. As TRs are functionally similar to the ecdysone receptor [59,60], and TRb expression in the chick retina is spatially and temporally similar to that of Shh, there is a possibility that TRb and Shh may be coordinated in a manner similar to that in the Drosophila retina. Beyond this potential relationship revealed by the spatio-temporal gradient of expression, other developmental regulators appear in central-peripheral waves in the chick retina, including Cath5, Otx2, NeuroD, Dio3, Dio2, Ngn2 and markers of various differentiated cell types. The mechanism(s) regulating these patterns is currently unknown.

### Cell Types Expressing TH Components

**Figure 10:**
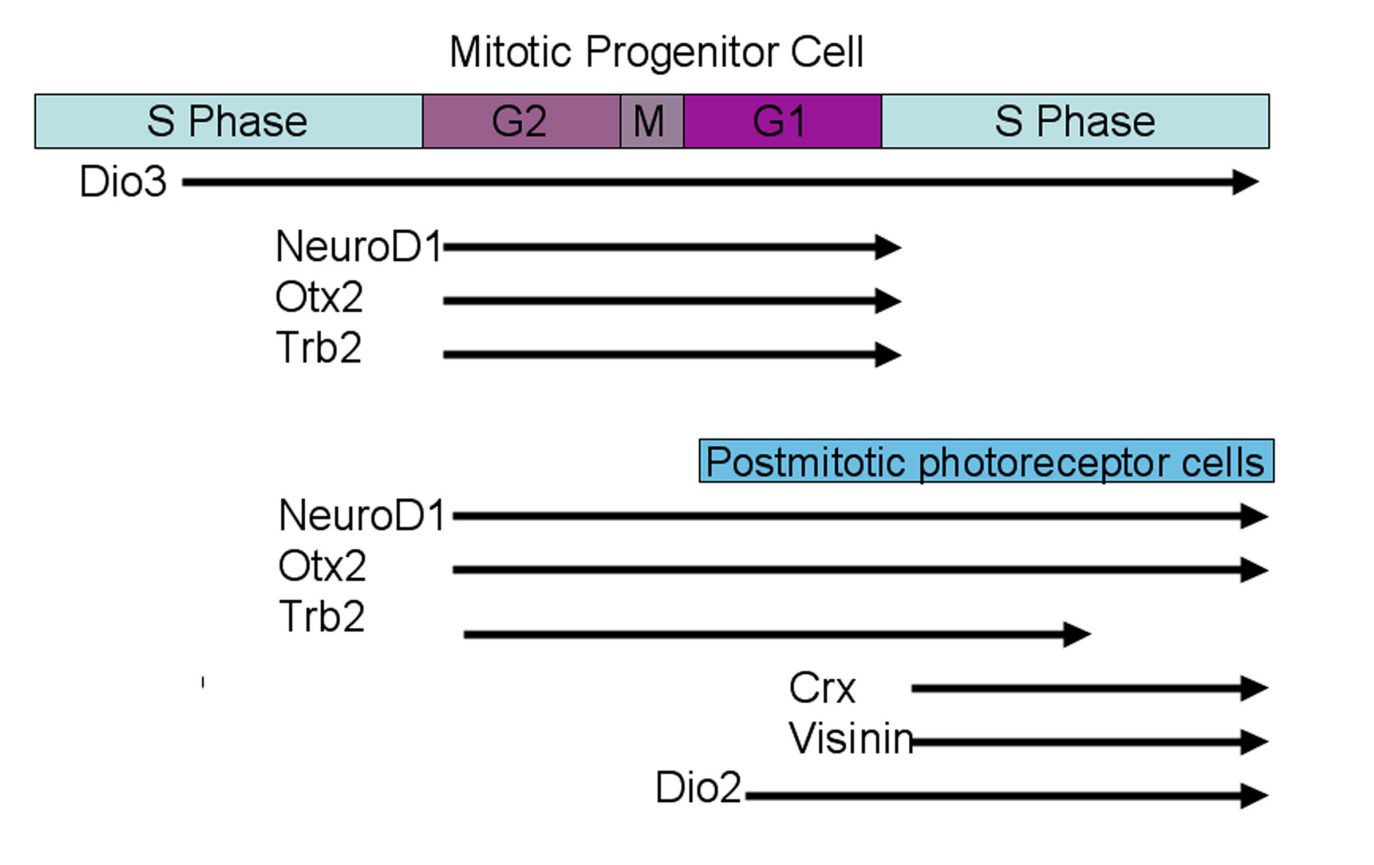
**Model of TH signaling in retinal development. **In mitotic progenitor cells, Dio3 is expressed throughout the cell cycle at various periods of development. In mitotic cells that are about to produce photoreceptor cells, the indicated genes are expressed in G2. As the cell divides, it is likely that one daughter of the division becomes a photoreceptor cell, and retains expression of TRb for some time, while the other daughter re-enters cell cycle and quickly turns off TRb. In photoreceptor cells, as TRb expression decreases, Crx and visinin are expressed, and a small subset of cells, possibly rods, transiently expresses Dio2.

The expression of TRb with respect to cell cycle kinetics and markers of photoreceptor cells show that this receptor is expressed in mitotic cells about to produce a postmitotic cell that will become a photoreceptor (Figure 10). It appears that such progenitor cells express Otx2 and NeuroD at almost exactly the same time as TRb. That the expression is in a G2 phase progenitor cell is an intriguing observation in light of retinal lineage data from embryonic mouse [61]. Infections of embryonic mice *in utero *with retroviral vectors were carried out at equivalent developmental stages to chick stages 26–32 when cones and rods are being produced in both species [62-64]. The majority of embryonic mouse retinal clones of >1 cell were not 2 cell clones, and this included the majority of clones that contained rods and cones. This observation suggests that when a postmitotic cell is made, it is not from a symmetric division in which both daughter cells become postmitotic, or many 2 cell clones would have been observed. If a similar result were to hold for the chick, the expression of TRb in G2 cells would not be maintained in both daughters of that division. Presumably, the postmitotic daughter would retain expression, at least transiently, as it initiated its differentiation into a photoreceptor cell. The other daughter would extinguish the expression of TRb as it re-entered S phase, as very few S phase cells express TRb. The expression of Otx2 and NeuroD is also likely maintained in the photoreceptor cell, as these genes appear to be expressed in the area of differentiating photoreceptor cells. They may also continue to be expressed in some of the S phase daughter cells, as there is higher number of S phase cells that express Otx2 and NeuroD, particularly at later stages.

It is interesting to note that Dio2 is expressed in a subset of photoreceptor cells. The Dio2 subset can be defined in three ways. It is highest in the ventral retina, and is missing in a central spot and horizontal stripe. The only other similar expression pattern is that of rhodopsin, a gene marking rod photoreceptors [28], which does not commence until Stage 44, 12 days after the Dio2 expression wanes. A second way to define the subset of Dio2^+ ^cells is by the time at which Dio2 appears in development, appearing later than the expression of Otx2, NeuroD, and TRb. Rod photoreceptors also appear to be generated later than cone photoreceptors in most species [14], although this has not been defined for the chick. Finally, the kinetics of expression of Dio2 in the cell cycle provides a third way to define it. Dio2 appears after the earlier expression of Otx2, NeuroD, and TRb cells in the subset of cells that express it. From all of these observations, it is possible that Dio2 is in rod photoreceptors. Confirmation of this hypothesis will await an early marker of rod photoreceptors in the chick as Dio2 disappears before expression of definitive rod-specific markers, such as rhodopsin.

### Model of TH Signaling in retinal development

The first wave of expression of TH components is characterized by mitotic cells transitioning from producing only mitotic daughters to a phase in which they begin to produce neurons. TRa appears to be the receptor present in all cells as they make this transition. Dio3 is present in progenitor cells at certain times, whereas TRb is expressed in some G2 cells and in newly postmitotic cells that begin to differentiate into photoreceptor cells. Based on these observations, it is possible that Dio3 keeps TRa cells in the state where they only generate mitotic daughter cells. When Dio3 expression ceases, the TRa cells can produce a postmitotic cell, and some of these daughter cells express TRb.

Whether Dio3 promotes cell division and blocks differentiation remains an unanswered question. Studies in the *Xenopus *retina during metamorphosis seem to challenge this notion, because in these experiments, Dio3 blocked cell division and antagonism of Dio3 promoted cell division [20]. However, these studies measured BrdU incorporation over a span of many days after manipulation of Dio3. The increased BrdU incorporation seen after antagonizing Dio3 could be due to several mechanisms, including an increase in cells undergoing their last cell division. This interpretation would agree with Dio3 promoting the progenitor cell state and a lack of Dio3 promoting cell cycle exit.

The second wave of expression of TH components is characterized by the maturation and migration of certain cell types to their final positions. Before the second wave, TRb photoreceptors appear to be in an immature photoreceptor state. During wave 2, they progress in their differentiation, as shown by expression of Crx. Locally activated TH might influence TRb cells during this period. The second wave ends with Dio3 sweeping across the retina, which might halt TRa cells from producing postmitotic cells which express TRb, and might instead change the types of cells produced, or the balance of mitotic and postmitotic daughters made by TRa progenitor cells.

A model consistent with this expression pattern is that TRb, in conjunction with TH produced by Dio2, serves to block photoreceptor differentiation. In this model, TRb appears in the early photoreceptor just as it is being produced, significantly before the cell expresses Crx. Eventually, TRb and/or Dio2 levels are reduced and the cell is allowed to continue differentiation and express Crx. In the ventral retina, where retinal Dio2 is present, the delay may be longer, which might contribute to the development of rod photoreceptors in this area. This repressive activity of TRb in the presence of Dio2 might be similar to TRb activity in other parts of the nervous system, including the inhibitory activity of TRb in the presence of T3 on transcription of the TSHb gene [65,66].

Previous data from mice support a repression and activation role of TRb in retinal photoreceptors. Loss of TRb led to cones that appeared to differentiate early, as seen by early S-opsin expression. Interestingly, in the same mutant, M-opsin failed to be expressed. Recently, it was shown that mutation of TRb2 to an allele that could not bind TH exhibited both aspects of the phenotype, demonstrating that both repression of S-opsin and activation of M-opsin requires TH binding [48,67]. In addition, TH was found at higher levels in the dorsal than the ventral retina [67], and Dio2 was found to be expressed at higher levels in the dorsal than in the ventral retina [68]. Thus, the coordination of TH levels through Dio2 activity, and the activity of the TRb2, controls at least one aspect of the dorsoventral gradient of both S and M-opsins. NeuroD was also shown to play a role in regulation of S and M-opsins, at least in part via its regulation of TRb2 [69]. Mice deficient in NeuroD showed the same S and M-opsin phenotypes as the TRb2 mutant, along with a lack of maintenance of Trb2 expression. NeuroD was shown to bind directly to a regulatory element for TRb2 [70], but was not itself sufficient to activate TRb2, in keeping with the observation that many more cells express NeuroD than express TRb2 (Table 1, 2, Figure 8; and [34]). It is interesting to note that in the chick, NeuroD expression is concomitant with TRb2 expression in its onset, but at least in the mouse, the onset of expression of TRb2 occurred in the NeuroD mutant, but was not maintained.

Finally, the third wave of expression of TH components is characterized by waning Dio3 expression and the transition from progenitor cells to Müller glial cells. Similar to the models described above, Dio3 at this stage may keep TRa cells in the progenitor state. When Dio3 expression ends, the TRa cells may be free to exit cell cycle and begin differentiating. However, at this late stage, cells may no longer be competent to generate photoreceptors. Instead, they may exit cell cycle and become Notch^+ ^Müller glia cells. This latter suggestion follows the observations of TH signaling in optic nerve cell cultures where TH has been demonstrated to promote the differentiation of oligodendrocyte cells [71-74]. In this well studied system, TH can promote the exit of oligodendrocyte precursor cells from the cell cycle and initiate differentiation. This action appears to be through TRa [75]. Lack of TH can block this process, promoting the expression of cell cycle genes and keeping cells in the proliferative state.

## Conclusion

In summary, TH components are expressed in dynamic waves across the developing chick retina, and involve a number of cell types including progenitor cells and photoreceptors. Through the expression of deiodinases that activate and degrade thyroid hormone, in conjunction with cells expressing TRs, a complex interplay between the different TH components likely results. An attractive hypothesis is that the hormone controls the timing of cell cycle exit for both photoreceptors and Müller glia cells, as well as the timing of differentiation for photoreceptors. Future functional studies will shed more light on these proposed activities.

## Methods

### Chick Embryos

Fertilized White Leghorn eggs (SPAFAS, Norwich, CT) were incubated at 38°C. Embryos were staged as in Hamburger and Hamilton [76].

### In situ hybridizations

Section *in situ *hybridization on 20 μm retinal cryosections was performed as previously described [77]. Flat-mount *in situ *hybridization was performed as previously described [28] with some modifications [78]. The chicken *in situ *probes used were based in the coding regions, except for one of the Dio3 probes and the Glut1 probe that were located at the 3' end. The numbering of the probe sequences was derived from the full length cDNA clones in the NCBI database. The exact regions that the probes spanned were as follows: [Dio2 (bases 596–1491)[79], TRa (bases 289–1538)[80], TRb (bases 42–1351)[15], Dio3 (probe #1, bases 872–1025 and probe #2 (chEST268e4), bases 30–792 [81]), Crx (bases 1–1296), NeuroD (bases 25–1162), Otx2 (bases 698–2001), Shh (bases 1–1567), Cath5 (bases 1456–1776), Glut1 (bases 2530–3203), Brn3b (bases 53–210)[39], and Visinin (bases 143–393)].

### [^3^H] thymidine – labeling and dissociated cell in situ hybridization

Chick retinas were labeled *in ovo *by the addition of 100 μCi of [^3^H]-thymidine and the retinas were dissected at the indicated timepoints. The labeled retinas were dissociated and plated on poly-D lysine coated slides (10 μg/ml in PBS [Sigma]) exactly as described in [77]. Cells were fixed to the slides with 4% paraformaldehyde (PFA) for 10 min. at room temperature, washed in PBS (pH 7.4) and dehydrated into 100% methanol. At this point, the slides were either stored at -20°C or rehydrated stepwise into PBS (pH 7.4) to continue the ISH. The slides were acetylated and incubated with single probes or distinct probe combinations overnight at 65°C. The probed slides were washed once in 5× SSC and an additional two times in 0.2× SSC for 30 min. each at 65°C. Slides were blocked in 0.1 M Tris-HCl, pH 7.5, 0.15 M NaCl and 10% heat inactivated sheep serum (HISS) for 30 min. at room temperature in a humidified chamber. In the identical chamber, the slides were incubated with the blocking solution containing anti-digoxigenin-POD (1:1000, Roche) for 1 hr. The slides were washed 3 times in 0.1 M Tris-HCl, pH 7.5, 0.15 M NaCl, 0.05% Tween-20 for 10 min. each. The first probe, which was labeled with digoxigenin, was processed with a Cy3 tyramide amplification solution (1:50, PerkinElmer Life Sciences) for 5 min. After quenching with hydrogen peroxide for 15 min., the second probe, which was labeled with fluorescein, was detected as above using an anti-fluorescein-POD antibody (1:1000, Roche) in combination with an Alexa 488-tyramide (1:100, Molecular Probes). The slides were washed in PBS (pH 7.4), stained with DAPI, and then allowed to dry. To visualize the [^3^H]-thymidine, slides were dipped in an autoradiography emulsion (NTB2, Kodak) and exposed in the dark for 2 days. The slides were subsequently immersed in developer for 2 min. (D19, Kodak), rinsed in dH_2_O, and incubated in fixer (Kodak) for 20 min. Finally, the slides were washed in dH_2_O for 20 min. and mounted.

For Stage 26 and Stage 32, the results are the average of 3 retinas, except for the 6 hr timepoint at Stage 26, which is the average of 2 retinas. For the other stages, the data were generated from a single retina for each time point.

## Authors' contributions

JMT designed and carried out the dissociated cell in situ hybridizations, performed some of the section in situ hybridizations and helped write the manuscript. SH designed and carried out some of the section in situ hybridizations, performed the flat mount in situ hybridizations and helped write the manuscript. NAB carried out the in situ hybridizations in Figure 2 and helped write the manuscript. CLC designed and coordinated the experiments and helped to write the manuscript.

## Supplementary Material

Additional file 1 **The kinetics of expression of TH components and markers at Stage 26.** Cells were continuously labeled in ovo with [^3^H]-thymidine, and tissue was harvested and dissociated. The total [^3^H]-thymidine labeling time is indicated after the gene name in the table. DISH for the indicated genes and autoradiography for [^3^H] were carried out on the dissociated cells. Click here for file

Additional file 2 **The kinetics of expression of TH components and markers at Stage 28.** Cells were continuously labeled in ovo with [^3^H]-thymidine, and tissue was harvested and dissociated. The total [^3^H]-thymidine labeling time is indicated after the gene name in the table. DISH for the indicated genes and autoradiography for [^3^H] were carried out on the dissociated cells. Click here for file

Additional file 3 **Coexpression of TH components and known marker genes.** Retinas were harvested at Stage 32 and dissociated onto glass slides. The slides were probed with (A-C) TRb and Otx2, (D-F) TRb and Crx, (G-I), Dio2 and TRb, (J-L) Dio2 and Crx, and (M-O) Dio2 and Dio3. The scale bar indicates 25 μm. Click here for file

Additional file 4 **Overlap between TH components or known marker genes and [^3^H]-thymidine.** Cells were labeled in ovo with [^3^H]-thymidine at Stage 26, Stage 28 or Stage 32, and the retinas were harvested and dissociated at the indicated times. DISH was carried for the following probes: (A-D) Otx2, (E-H) TRb, (I-L) Crx, (M-P) Dio3, and (Q-R) Dio2. Autoradiography was performed to visualize the [^3^H]-thymidine and the overlap with the DISH is shown. The scale bar indicates 25 μm. Click here for file

## References

[B1] Brown DD (2005). The role of deiodinases in amphibian metamorphosis. Thyroid.

[B2] Park SM (2005). VK Chatterjee: Genetics of congenital hypothyroidism. J Med Genet.

[B3] Porterfield SP (1993). CE Hendrich: The role of thyroid hormones in prenatal and neonatal neurological development – current perspectives. Endocr Rev.

[B4] Lazar MA (1990). WW Chin: Nuclear thyroid hormone receptors. J Clin Invest.

[B5] Hu X, Lazar MA (2000). Transcriptional repression by nuclear hormone receptors. Trends Endocrinol Metab.

[B6] Zhang J, Lazar MA (2000). The mechanism of action of thyroid hormones. Annu Rev Physiol.

[B7] Nishihara E, O'Malley BW, Xu J (2004). Nuclear receptor coregulators are new players in nervous system development and function. Mol Neurobiol.

[B8] Bianco AC, Larsen PR (2005). Cellular and structural biology of the deiodinases. Thyroid.

[B9] Bianco AC, Kim BW (2006). Deiodinases: implications of the local control of thyroid hormone action. J Clin Invest.

[B10] Galton VA (2005). The roles of the iodothyronine deiodinases in mammalian development. Thyroid.

[B11] Campos-Barros A, Amma LL, Faris JS, Shailam R, Kelley MW, Forrest D (2000). Type 2 iodothyronine deiodinase expression in the cochlea before the onset of hearing. Proc Natl Acad Sci USA.

[B12] Mai W, Janier MF, Allioli N, Quignodon L, Chuzel T, Flamant F, Samarut J (2004). Thyroid hormone receptor alpha is a molecular switch of cardiac function between fetal and postnatal life. Proc Natl Acad Sci USA.

[B13] Livesey FJ, Cepko CL (2001). Vertebrate neural cell-fate determination: lessons from the retina. Nat Rev Neurosci.

[B14] Altshuler DM, Turner DL, Cepko CL (1991). Specification of cell type in the vertebrate retina. Development of the Visual System.

[B15] Forrest D, Sjoberg M, Vennstrom B (1990). Contrasting developmental and tissue-specific expression of alpha and beta thyroid hormone receptor genes. Embo J.

[B16] Forrest D, Hallbook F, Persson H, Vennstrom B (1991). Distinct functions for thyroid hormone receptors alpha and beta in brain development indicated by differential expression of receptor genes. Embo J.

[B17] Sjoberg M, Vennstrom B, Forrest D (1992). Thyroid hormone receptors in chick retinal development: differential expression of mRNAs for alpha and N-terminal variant beta receptors. Development.

[B18] Sevilla-Romero E, Munoz A, Pinazo-Duran MD (2002). Low thyroid hormone levels impair the perinatal development of the rat retina. Ophthalmic Res.

[B19] Hoskins SG (1990). Metamorphosis of the amphibian eye. J Neurobiol.

[B20] Marsh-Armstrong N, Huang H, Remo BF, Liu TT, Brown DD (1999). Asymmetric growth and development of the Xenopus laevis retina during metamorphosis is controlled by type III deiodinase. Neuron.

[B21] Hoar W (1988). Fish Physiology.

[B22] Browman HI, Hawryshyn CW (1994). The developmental trajectory of ultraviolet photosensitivity in rainbow trout is altered by thyroxine. Vision Res.

[B23] Allison WT, Dann SG, Veldhoen KM, Hawryshyn CW (2006). Degeneration and regeneration of ultraviolet cone photoreceptors during development in rainbow trout. J Comp Neurol.

[B24] Kelley MW, Turner JK, Reh TA (1995). Ligands of steroid/thyroid receptors induce cone photoreceptors in vertebrate retina. Development.

[B25] Kelley MW, Turner JK, Reh TA (1995). Regulation of proliferation and photoreceptor differentiation in fetal human retinal cell cultures. Invest Ophthalmol Vis Sci.

[B26] Ng L, Hurley JB, Dierks B, Srinivas M, Salto C, Vennstrom B, Reh TA, Forrest D (2001). A thyroid hormone receptor that is required for the development of green cone photoreceptors. Nat Genet.

[B27] Dutting D, Gierer A, Hansmann G (1983). Self-renewal of stem cells and differentiation of nerve cells in the developing chick retina. Brain Res.

[B28] Bruhn SL, Cepko CL (1996). Development of the pattern of photoreceptors in the chick retina. J Neurosci.

[B29] McCabe KL, Gunther EC, Reh TA (1999). The development of the pattern of retinal ganglion cells in the chick retina: mechanisms that control differentiation. Development.

[B30] Cho SH, Cepko CL (2006). Wnt2b/beta-catenin-mediated canonical Wnt signaling determines the peripheral fates of the chick eye. Development.

[B31] Bovolenta P, Mallamaci A, Briata P, Corte G, Boncinelli E (1997). Implication of OTX2 in pigment epithelium determination and neural retina differentiation. J Neurosci.

[B32] Roztocil T, Matter-Sadzinski L, Alliod C, Ballivet M, Matter JM (1997). NeuroM, a neural helix-loop-helix transcription factor, defines a new transition stage in neurogenesis. Development.

[B33] Liu W, Mo Z, Xiang M (2001). The Ath5 proneural genes function upstream of Brn3 POU domain transcription factor genes to promote retinal ganglion cell development. Proc Natl Acad Sci USA.

[B34] Morrow EM, Furukawa T, Lee JE, Cepko CL (1999). NeuroD regulates multiple functions in the developing neural retina in rodent. Development.

[B35] Ahmad I, Acharya HR, Rogers JA, Shibata A, Smithgall TE, Dooley CM (1998). The role of NeuroD as a differentiation factor in the mammalian retina. J Mol Neurosci.

[B36] Yan RT, Wang SZ (1998). neuroD induces photoreceptor cell overproduction in vivo and de novo generation in vitro. J Neurobiol.

[B37] Gan L, Xiang M, Zhou L, Wagner DS, Klein WH, Nathans J (1996). POU domain factor Brn-3b is required for the development of a large set of retinal ganglion cells. Proc Natl Acad Sci USA.

[B38] Xiang M (1998). Requirement for Brn-3b in early differentiation of postmitotic retinal ganglion cell precursors. Dev Biol.

[B39] Liu W, Khare SL, Liang X, Peters MA, Liu X, Cepko CL, Xiang M (2000). All Brn3 genes can promote retinal ganglion cell differentiation in the chick. Development.

[B40] Furukawa T, Morrow EM, Cepko CL (1997). Crx, a novel otx-like homeobox gene, shows photoreceptor-specific expression and regulates photoreceptor differentiation. Cell.

[B41] Zhang XM, Yang XJ (2001). Regulation of retinal ganglion cell production by Sonic hedgehog. Development.

[B42] Shkumatava A, Fischer S, Muller F, Strahle U, Neumann CJ (2004). Sonic hedgehog, secreted by amacrine cells, acts as a short-range signal to direct differentiation and lamination in the zebrafish retina. Development.

[B43] Dakubo GD, Wang YP, Mazerolle C, Campsall K, McMahon AP, Wallace VA (2003). Retinal ganglion cell-derived sonic hedgehog signaling is required for optic disc and stalk neuroepithelial cell development. Development.

[B44] Jadhav AP, Cho SH, Cepko CL (2006). Notch activity permits retinal cells to progress through multiple progenitor states and acquire a stem cell property. Proc Natl Acad Sci USA.

[B45] Edqvist PH, Hallbook F (2004). Newborn horizontal cells migrate bi-directionally across the neuroepithelium during retinal development. Development.

[B46] Bergersen L, Johannsson E, Veruki ML, Nagelhus EA, Halestrap A, Sejersted OM, Ottersen OP (1999). Cellular and subcellular expression of monocarboxylate transporters in the pigment epithelium and retina of the rat. Neuroscience.

[B47] Kumagai AK, Glasgow BJ, Pardridge WM (1994). GLUT1 glucose transporter expression in the diabetic and nondiabetic human eye. Invest Ophthalmol Vis Sci.

[B48] Applebury ML, Farhangfar F, Glosmann M, Hashimoto K, Kage K, Robbins JT, Shibusawa N, Wondisford FE, Zhang H (2007). Transient expression of thyroid hormone nuclear receptor TRbeta2 sets S opsin patterning during cone photoreceptor genesis. Dev Dyn.

[B49] Fujita S, Horii M (1963). Analysis Of Cytogenesis In Chick Retina By Tritiated Thymidine Autoradiography. Arch Histol Jpn.

[B50] Kahn AJ (1974). An autoradiographic analysis of the time of appearance of neurons in the developing chick neural retina. Dev Biol.

[B51] Polans AS, Burton MD, Haley TL, Crabb JW, Palczewski K (1993). Recoverin, but not visinin, is an autoantigen in the human retina identified with a cancer-associated retinopathy. Invest Ophthalmol Vis Sci.

[B52] Ready DF, Hanson TE, Benzer S (1976). Development of the Drosophila retina, a neurocrystalline lattice. Dev Biol.

[B53] Neumann CJ, Nuesslein-Volhard C (2000). Patterning of the zebrafish retina by a wave of sonic hedgehog activity. Science.

[B54] Perron M, Boy S, Amato MA, Viczian A, Koebernick K, Pieler T, Harris WA (2003). A novel function for Hedgehog signalling in retinal pigment epithelium differentiation. Development.

[B55] Matter-Sadzinski L, Puzianowska-Kuznicka M, Hernandez J, Ballivet M, Matter JM (2005). A bHLH transcriptional network regulating the specification of retinal ganglion cells. Development.

[B56] Ma C, Zhou Y, Beachy PA, Moses K (1993). The segment polarity gene hedgehog is required for progression of the morphogenetic furrow in the developing Drosophila eye. Cell.

[B57] Heberlein U, Wolff T, Rubin GM (1993). The TGF beta homolog dpp and the segment polarity gene hedgehog are required for propagation of a morphogenetic wave in the Drosophila retina. Cell.

[B58] Brennan CA, Ashburner M, Moses K (1998). Ecdysone pathway is required for furrow progression in the developing Drosophila eye. Development.

[B59] Evans R (2004). A transcriptional basis for physiology. Nat Med.

[B60] Mangelsdorf DJ, Thummel C, Beato M, Herrlich P, Schutz G, Umesono K, Blumberg B, Kastner P, Mark M, Chambon P (1995). The nuclear receptor superfamily: the second decade. Cell.

[B61] Turner DL, Snyder EY, Cepko CL (1990). Lineage-independent determination of cell type in the embryonic mouse retina. Neuron.

[B62] Prada C, Puga J, Perez-Mendez L, Lopez R, Ramirez G (1991). Spatial and Temporal Patterns of Neurogenesis in the Chick Retina. Eur J Neurosci.

[B63] Belecky-Adams T, Cook B, Adler R (1996). Correlations between terminal mitosis and differentiated fate of retinal precursor cells in vivo and in vitro: analysis with the "window-labeling" technique. Dev Biol.

[B64] Carter-Dawson LD, LaVail MM (1979). Rods and cones in the mouse retina. II. Autoradiographic analysis of cell generation using tritiated thymidine. J Comp Neurol.

[B65] Chin WW, Carr FE, Burnside J, Darling DS (1993). Thyroid hormone regulation of thyrotropin gene expression. Recent Prog Horm Res.

[B66] Matsushita A, Sasaki S, Kashiwabara Y, Nagayama K, Ohba K, Iwaki H, Misawa H, Ishizuka K, Nakamura H (2007). Essential role of GATA2 in the negative regulation of thyrotropin beta gene by thyroid hormone and its receptors. Mol Endocrinol.

[B67] Roberts MR, Srinivas M, Forrest D, Morreale de Escobar G, Reh TA (2006). Making the gradient: thyroid hormone regulates cone opsin expression in the developing mouse retina. Proc Natl Acad Sci USA.

[B68] Corbo JC, Myers CA, Lawrence KA, Jadhav AP, Cepko CL (2007). A typology of photoreceptor gene expression patterns in the mouse. Proc Natl Acad Sci USA.

[B69] Liu H, Etter P, Hayes S, Jones I, Nelson B, Hartman B, Forrest D, Reh TA (2008). NeuroD1 regulates expression of thyroid hormone receptor 2 and cone opsins in the developing mouse retina. J Neurosci.

[B70] Jones I, Ng L, Liu H, Forrest D (2007). An intron control region differentially regulates expression of thyroid hormone receptor beta2 in the cochlea, pituitary, and cone photoreceptors. Mol Endocrinol.

[B71] Billon N, Tokumoto Y, Forrest D, Raff M (2001). Role of thyroid hormone receptors in timing oligodendrocyte differentiation. Dev Biol.

[B72] Ahlgren SC, Wallace H, Bishop J, Neophytou C, Raff MC (1997). Effects of thyroid hormone on embryonic oligodendrocyte precursor cell development in vivo and in vitro. Mol Cell Neurosci.

[B73] Barres BA, Lazar MA, Raff MC (1994). A novel role for thyroid hormone, glucocorticoids and retinoic acid in timing oligodendrocyte development. Development.

[B74] Durand B, Raff M (2000). A cell-intrinsic timer that operates during oligodendrocyte development. Bioessays.

[B75] Billon N, Jolicoeur C, Tokumoto Y, Vennstrom B, Raff M (2002). Normal timing of oligodendrocyte development depends on thyroid hormone receptor alpha 1 (TRalpha1). Embo J.

[B76] Hamburger V, Hamilton HL (1992). A series of normal stages in the development of the chick embryo. 1951. Dev Dyn.

[B77] Trimarchi JM, Stadler MB, Roska B, Billings N, Sun B, Bartch B, Cepko CL (2007). Molecular heterogeneity of developing retinal ganglion and amacrine cells revealed through single cell gene expression profiling. J Comp Neurol.

[B78] Chen CM, Cepko CL (2002). The chicken RaxL gene plays a role in the initiation of photoreceptor differentiation. Development.

[B79] Gereben B, Bartha T, Tu HM, Harney JW, Rudas P, Larsen PR (1999). Cloning and expression of the chicken type 2 iodothyronine 5'-deiodinase. J Biol Chem.

[B80] Sap J, Munoz A, Damm K, Goldberg Y, Ghysdael J, Leutz A, Beug H, Vennstrom B (1986). The c-erb-A protein is a high-affinity receptor for thyroid hormone. Nature.

[B81] Boardman PE, Sanz-Ezquerro J, Overton IM, Burt DW, Bosch E, Fong WT, Tickle C, Brown WR, Wilson SA, Hubbard SJ (2002). A comprehensive collection of chicken cDNAs. Curr Biol.

