# Supplementary material for: Thyroid hormone components are expressed in three sequential waves during development of the chick retina
Source: BMC Dev Biol. 2008 Oct 14;8:101. doi: 10.1186/1471-213X-8-101 (PMC2579430; doi:10.1186/1471-213X-8-101)
Supplement: Additional file 1 — The kinetics of expression of TH components and markers at Stage 26. Cells were continuously labeled in ovo with [3H]-thymidine, and tissue was harvested and dissociated. The total [3H]-thymidine labeling time is indicated after the gene name in the table. DISH for the indicated genes and autoradiography for [3H] were carried out on the dissociated cells. [file 1471-213X-8-101-S1.doc]

# Kinetics of expression of TH components and markers at stage 26

| **gene** | **% gene^+^ cells** | **% [^3^H]^+^** | **% of gene^+^cells that are [^3^H]^+^** | **% of [^3^H]^+^cells that are gene^+^** |
| --- | --- | --- | --- | --- |

| **TRb_1hr** | **6.0 ± 0.2** | **37.6 ± 1.5** | **16.9 ± 3.8** | **2.8 ± 0.7** |
| --- | --- | --- | --- | --- |
| **TRb _2hr** | **8.1 ± 0.7** | **38.4 ± 2.1** | **18.9 ± 6.9** | **3.9 ± 1.3** |
| **TRb _4hr** | **8.7 ± 0.8** | **65.9 ± 0.5** | **35.8 ± 4.2** | **4.7 ± 0.6** |
| **TRb _6hr** | **7.3** | **84.8** | **67.2** | **5.8** |
| **TRb _8hr** | **7.7 ± 0.4** | **90.2 ± 0.9** | **77.4 ± 1.6** | **6.7 ± 0.4** |
| **otx2_1hr** | **8.8 ± 0.4** | **42.7 ± 1.8** | **19.5 ± 6.3** | **4.2 ± 1.5** |
| **otx2_2hr** | **12.8** | **38.5** | **25.2** | **8.4** |
| **otx2_4hr** | **11.8 ± 0.8** | **67.1 ±1.2** | **37.9 ± 4.9** | **6.6 ± 0.9** |
| **otx2_6hr** | **8.6** | **87.9** | **66.3** | **6.5** |
| **otx2_8hr** | **9.1 ± 0.8** | **90.3 ± 1.7** | **83.4 ± 4.9** | **8.4 ± 0.8** |
| **neurod_1hr** | **9.7 ± 1.0** | **44.6 ± 1.7** | **23.2 ± 0.7** | **5.1 ± 0.6** |
| **neurod_2hr** | **17.8 ± 3.4** | **38.1 ± 2.1** | **17.8 ± 1.5** | **8.1 ± 1.1** |
| **neurod_4hr** | **10.2 ± 1.3** | **65.7 ± 2.8** | **42.8 ± 5.0** | **6.9 ± 1.6** |
| **neurod_6hr** | **17.1** | **84.9** | **83.4** | **16.8** |
| **neurod_8hr** | **14.2 ± 0.8** | **90.6 ± 0.7** | **84.5 ± 2.6** | **13.2 ± 0.4** |

**Dio2, Dio3 and Crx were each undetectable (<1/1000 cells) at stage 26.**

# 
