# Supplementary material for: Thyroid hormone components are expressed in three sequential waves during development of the chick retina
Source: BMC Dev Biol. 2008 Oct 14;8:101. doi: 10.1186/1471-213X-8-101 (PMC2579430; doi:10.1186/1471-213X-8-101)
Supplement: Additional file 2 — The kinetics of expression of TH components and markers at Stage 28. Cells were continuously labeled in ovo with [3H]-thymidine, and tissue was harvested and dissociated. The total [3H]-thymidine labeling time is indicated after the gene name in the table. DISH for the indicated genes and autoradiography for [3H] were carried out on the dissociated cells. [file 1471-213X-8-101-S2.doc]

# Kinetics of expression of TH components and markers at stage 28

| **gene** | **% gene^+^ cells** | **% [^3^H]^+^** | **% of gene^+^cells that are [^3^H]^+^** | **% of [^3^H]^+^cells that are gene^+^** |
| --- | --- | --- | --- | --- |

| **TRb _1hr** | **11.8** | **34.2** | **13.6** | **4.7** |
| --- | --- | --- | --- | --- |
| **TRb _2hr** | **9.6** | **44.7** | **23.4** | **5** |
| **TRb _4hr** | **12.4** | **64.9** | **33** | **6.3** |
| **TRb _6hr** | **8.1** | **73.1** | **48.8** | **5.4** |
| **TRb _8hr** | **6.7** | **80.8** | **53.5** | **4.4** |
| **TRb _10hr** | **8.2** | **77** | **62.2** | **6.6** |
| **dio3_1hr** | **3.3** | **31** | **54.2** | **5.8** |
| **dio3_4hr** | **2.9** | **71.4** | **75** | **3** |
| **dio3_6hr** | **4.5** | **80.3** | **91.7** | **5.2** |
| **dio3_8hr** | **5** | **82.8** | **100** | **6.1** |
| **neurod_1hr** | **12.8** | **34** | **25.8** | **9.7** |
| **neurod_2hr** | **10.6** | **60.4** | **48** | **8.4** |
| **neurod_4hr** | **19** | **65.9** | **53.5** | **15.4** |
| **neurod_6hr** | **20.7** | **78.2** | **74.9** | **19.8** |
| **neurod_8hr** | **21.4** | **84.6** | **81.5** | **20.6** |
| **neurod_10hr** | **17.5** | **79.9** | **74.8** | **16.4** |
| **crx_1hr** | **5.9** | **32.5** | **0** | **0** |
| **crx_4hr** | **8.2** | **69.2** | **8.9** | **1.1** |
| **crx_8hr** | **6.7** | **83.9** | **28** | **2.2** |
