# Supplementary figures and images for: Thyroid hormone components are expressed in three sequential waves during development of the chick retina
Source: BMC Dev Biol. 2008 Oct 14;8:101. doi: 10.1186/1471-213X-8-101 (PMC2579430; doi:10.1186/1471-213X-8-101)

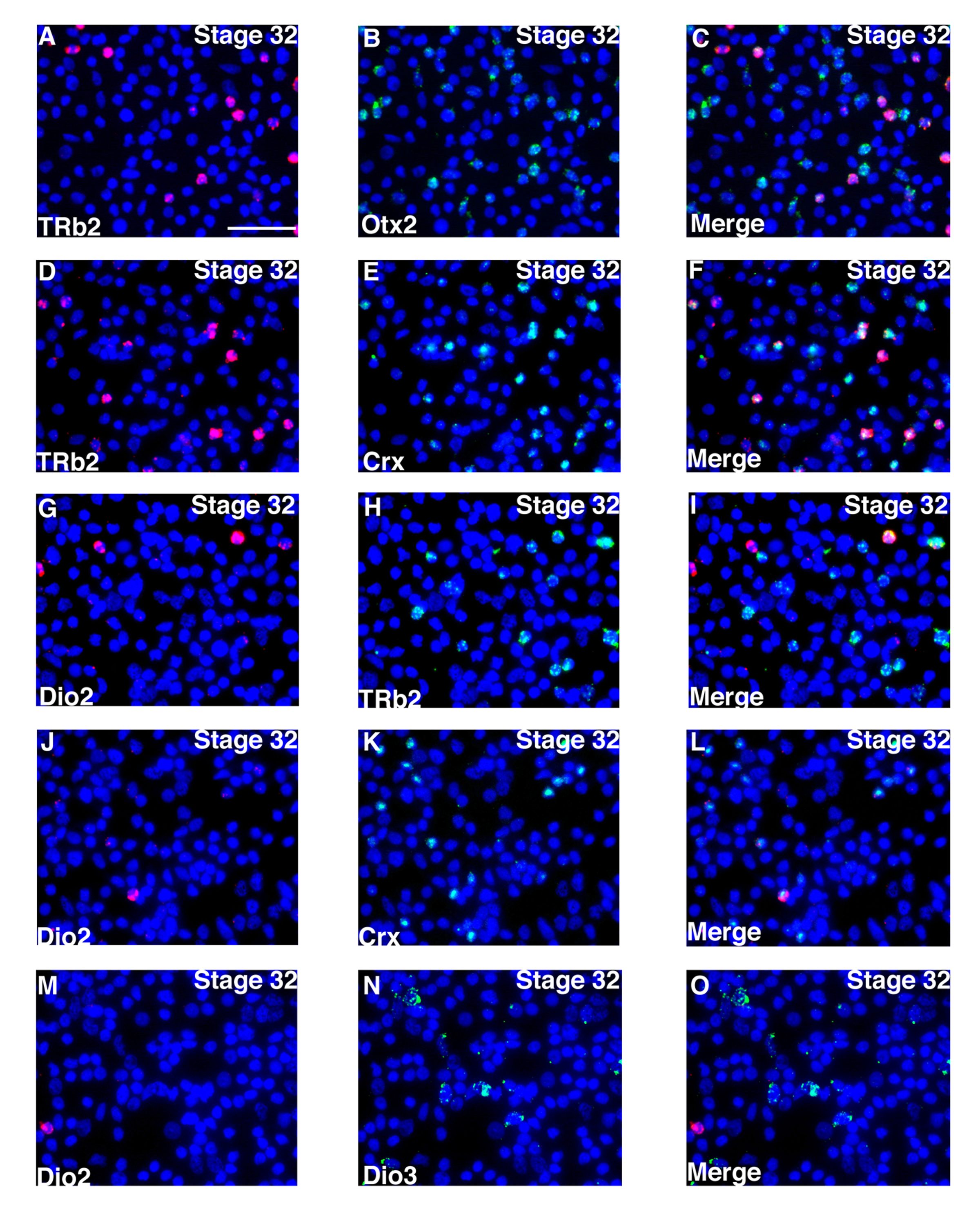

Supplement: Additional file 3 — Coexpression of TH components and known marker genes. Retinas were harvested at Stage 32 and dissociated onto glass slides. The slides were probed with (A-C) TRb and Otx2, (D-F) TRb and Crx, (G-I), Dio2 and TRb, (J-L) Dio2 and Crx, and (M-O) Dio2 and Dio3. The scale bar indicates 25 μm. [file 1471-213X-8-101-S3.tiff]

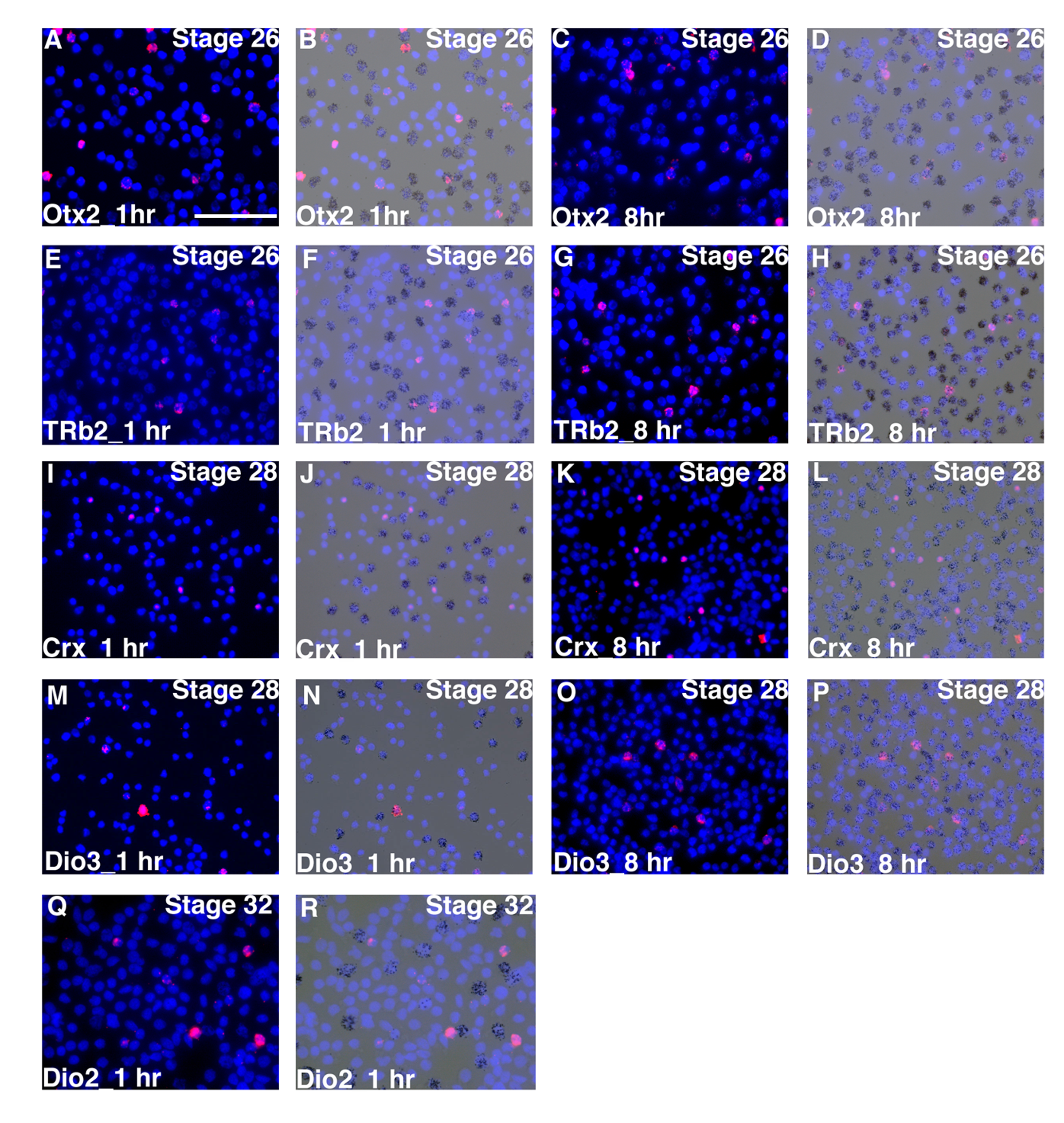

Supplement: Additional file 4 — Overlap between TH components or known marker genes and [3H]-thymidine. Cells were labeled in ovo with [3H]-thymidine at Stage 26, Stage 28 or Stage 32, and the retinas were harvested and dissociated at the indicated times. DISH was carried for the following probes: (A-D) Otx2, (E-H) TRb, (I-L) Crx, (M-P) Dio3, and (Q-R) Dio2. Autoradiography was performed to visualize the [3H]-thymidine and the overlap with the DISH is shown. The scale bar indicates 25 μm. [file 1471-213X-8-101-S4.tiff]
